# Empowering Tomorrow’s Cancer Specialists: Evaluating the Co-creation and Impact of Malawi’s First Surgical Oncology Summerschool

**DOI:** 10.1007/s13187-024-02400-5

**Published:** 2024-02-09

**Authors:** Remco van Egmond, Jurre van Kesteren, Lucy Kaomba, Godfrey Sama Philipo, Eric Borgstein, Inne Borel Rinkes, Marcus Rijken, Schelto Kruijff, Eva Stortelder

**Affiliations:** 1 Department of Surgery, Ziekenhuis Gelderse Vallei, Ede, The Netherlands; 2 Netherlands Society for International Surgery (NSIS), Soest, The Netherlands; 3 Department of Surgery, Amsterdam University Medical Centres, Location VUmc, Amsterdam, The Netherlands; 4 Global Surgery Amsterdam, Amsterdam, The Netherlands; 5 Department of Surgery, Kamuzu University of Health Sciences, Blantyre, Malawi; 6 Department of Surgery, Queen Elizabeth Central Hospital, Blantyre, Malawi; 7 The College of Surgeons of East Central and Southern Africa (COSECSA), Arusha, Tanzania; 8 The Branch for Global Surgical Care (BGSC), University of British Columbia, Vancouver, Canada; 9 Department of Oncology, Utrecht University Medical Centre, Utrecht, The Netherlands; 10 Netherlands Cancer Institute, Amsterdam, The Netherlands; 11 Workingparty International Safe Motherhood & Reproductive Health, Groningen, The Netherlands; 12 Department of OBGYN, Amsterdam University Medical Centre, Amsterdam, The Netherlands; 13 Julius Global Health, Utrecht University Medical Centre Utrecht, Utrecht, The Netherlands; 14 Department of Surgery, University Medical Centre, Groningen, The Netherlands; 15 Department of Surgery, Utrecht University Medical Centre, Utrecht, The Netherlands

**Keywords:** Surgical oncology education, Low resource settings, Cancer care in Sub-Saharan Africa, Kirkpatrick’s evaluation model

## Abstract

Annually more than 1 million newly diagnosed cancer cases and 500,000 cancer-related deaths occur in Sub Saharan Africa (SSA). By 2030, the cancer burden in Africa is expected to double accompanied by low survival rates. Surgery remains the primary treatment for solid tumours especially where other treatment modalities are lacking. However, in SSA, surgical residents lack sufficient training in cancer treatment. In 2022, Malawian and Dutch specialists co-designed a training course focusing on oncologic diseases and potential treatment options tailored to the Malawian context. The aim of this study was to describe the co-creation process of a surgical oncology education activity in a low resource setting, at the same time attempting to evaluate the effectiveness of this training program. The course design was guided and evaluated conform Kirkpatrick’s *requirements for an effective training program*. Pre-and post-course questionnaires were conducted to evaluate the effectiveness. Thirty-five surgical and gynaecological residents from Malawi participated in the course. Eighty-six percent of respondents (*n* = 24/28) were highly satisfied at the end of the course. After a 2-month follow-up, 84% (*n* = 16/19) frequently applied the newly acquired knowledge, and 74% (*n* = 14/19) reported to have changed their patient care. The course costs were approximately 119 EUR per attendee per day. This course generally received generally positively feedback, had high satisfaction rates, and enhanced knowledge and confidence in the surgical treatment of cancer. Its effectiveness should be further evaluated using the same co-creation model in different settings. Integrating oncology into the regular curriculum of surgical residents is recommended.

## Introduction

Cancer is the second leading cause of mortality globally, responsible for approximately 9.6 million deaths annually [1]. Most of these deaths (70%) occur in low- and middle-income countries (LMICs) [2]. Annually there are more than 1 million new oncology cases and 500,000 cancer-related deaths in Sub Saharan Africa (SSA). Without intervention, SSA may see cancer cases doubling by 2040 [3]. Surgical oncology is vital in cancer management, offering the greatest opportunity to restore health, alleviate pain, and extend life for patients, particularly in low-resource settings [4].

Malawi is one such example of a country facing significant challenges in diagnosing cancer, providing surgical treatment and support to patients when cure is not possible [5]. The country has limited medical resources and infrastructure, such as absence of radiotherapy, and a shortage of trained surgeons [6]. For health care professionals in Malawi and surrounding countries, currently, there is no separate curriculum in oncology. Providing short courses in surgical oncology may be a tool to educate local healthcare professionals in this field [7].

The International Summerschool Oncology for Medical Students (ISOMS) in Groningen, the Netherlands, is an example of a short course in oncology which has been annually organized since 1996 and described to increase general cancer care knowledge, reduce trainees' fear of communicating with cancer patients, and expose participants to cancer-related issues [8].

Despite being the largest surgical training institution in SSA, having trained over 900 surgeons, the College of Surgeons of East, Central and Southern Africa (COSECSA) has yet to formally incorporate surgical oncology as separate specialty into its training program. Currently, surgical oncology is embedded in the general surgery training [9]. Instead of sending trainees abroad, there are several advantages to hosting courses within the region. This approach offers ecological benefits by reducing travel, fosters context-based learning in participants' own clinical environments, lowers the participation threshold for local clinicians, and strengthens national and international oncological networks by involving local clinicians as facilitators.

To establish surgical oncology education for local surgical residents within COSECSA, a team of diverse specialists from Central Hospitals in Malawi collaborated with experienced surgical oncologists/teachers from the Netherlands, all with a background of working experience in the setting of SSA. Serving as a potential model for future courses, this study describes the co-creation process of a surgical oncology education in a low resource setting, at the same time attempting to evaluate the effectiveness of this training program.

## Methods

This study was conducted in Malawi, a low-income, landlocked country in south-eastern Africa with a population of approximately 19.9 million, where roughly 65% of the population lives on less than $1 per day [10]. Surgical care in Malawi is estimated to be provided by 370 healthcare workers, predominantly non-surgeons including medical doctors and clinical officers [11].

We present the development, implementation, and evaluation of a pilot course in surgical oncology held in Blantyre, in the proximity of Queen Elizabeth Central Hospital, which spanned 5 days from August 22–26, 2022. It focused on locally relevant surgical treatment options for various oncological conditions in the SSA context.

To successfully implement the training program and to evaluate its effectiveness, we used Kirkpatrick’s evaluation framework [12], which includes the determination of ideas, initial objectives, subject content, selecting participants, the best schedule, selecting a facility, and appropriate instructors, preparing audio visuals and coordinating and evaluating the program.

### Determining Ideas (01)

To address the increasing number of patients with oncological conditions and the limited training opportunities for residents, the surgical department of Kamuzu University of Health Sciences (KUHES) in Blantyre, Malawi, expressed the need for a short course in surgical oncology. One of the consultants approached the Netherlands Society for International Surgery for support in developing such a course.

### Setting Objectives (02)

This study aimed to develop a context-adapted short course in Malawi that covers prevalent oncological conditions and strengthens national, regional, and international oncological networks.

### Determining Subject Content (03)

The course outline and daily lectures were co-created by a team of specialists from Malawi and the Netherlands, combining global and local expertise to ensure relevance to the local context. Spanning 5 days, the course covered a range of oncologic subjects and diagnoses (Table 1). Topics were selected to facilitate basic oncologic education and based on cancer prevalence in the country, and therefore, gynaecological cancer was included in the program as well. Participants were requested to prepare and present a clinical case of an oncologic patient from their own practice, promoting contextually appropriate content and facilitating (inter)national networking opportunities among the lecturers and specialists in their respective fields.

**Table 1:**
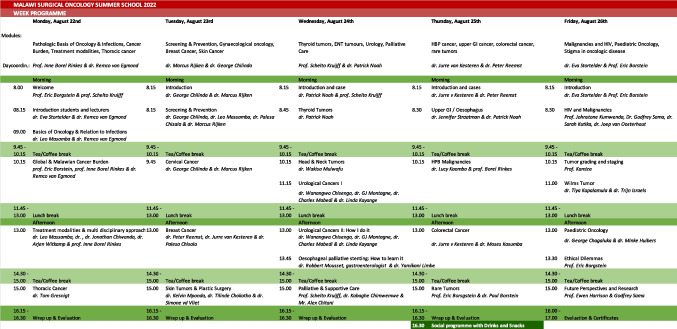
Full program short course in surgical oncology

### Selecting Participants (04)

The course targeted surgical and gynaecological residents from the COSECSA and KUHES programs. The local surgical team, led by the Malawian head of faculty, approached trainees. All COSECSA residents in Malawi were invited by email, with a provided flyer and course outline. Registration fees were waived with financial support from diverse sources, like foundations supporting oncologic education. Selected trainees received a daily allowance for expenses. The maximal number of participants was limited to 35, to allow interactive sessions, reduction of costs, and minimize impact on hospital capacity and ongoing quality of medical care in the country.

### Determining the Best Schedule (05)

Based on prevalence and available lecturers, a training schedule was co-designed with the Dutch-Malawian faculty. On day 1, oncological principals and the cancer prevalence in the Malawian context were introduced. Day 2 included prevention and screening, breast cancer, and oncologic gynaecology, involving gynaecology residents and consultant-level gynaecological oncologists, with a focus on cervical cancer. Day 3 covered head and neck tumours, oncologic urology, and palliative and supportive care. Day 4 focused on gastrointestinal tumours; day 5 addressed paediatric oncology, HIV and cancer, ethical dilemmas in cancer care, and conducting research.

### Selecting an Appropriate Facility (06)

We searched for a centrally located facility in Blantyre with conference rooms, lunch packages, and internet/Wi-Fi, enabling remote access to online tutorials. The course was held at a hotel in Blantyre, a few kilometres away from the main training site at Queen Elizabeth Central Hospital so lecturing clinicians were easily available.

### Selecting Appropriate Instructors (07)

The organizing committee and faculty for the course comprised a total of 40 specialists and residents, including from Malawi: four general surgeons, two urologists, one urological resident, three internal medicine specialists, two palliative care doctors, two paediatric surgeons, one dermatologist, one ENT specialist, one gynaecologist, one pathologist, one plastic surgeon, and one surgical resident; from the Netherlands, four surgical oncologists, three surgical residents, one gastroenterologist, one gastrointestinal surgeon, one general surgeon, one gynaecologist, one HPB surgeon, one paediatric oncologist, one paediatric and oncologic surgeon, one urologist, and one dermatologist; from Tanzania, one oncologist and one researcher in public health; from the UK, one HPB surgeon. The international surgical network of the Netherlands Society for International Surgery (NSIS), a working group under the Dutch Surgical Society, selected lecturers from high-income settings with a strong background in oncology and relevant work experience in low-resource settings. The working group International Safe Motherhood and Reproductive Health was responsible for the gynaecological input. Lecturers from low- and high-income settings were paired based on their specialization and jointly prepared their course presentations. From the Netherlands, two surgical residents, one gynaecologist, and one paediatric and oncologic surgeon participated onsite; the others contributed online.

### Selecting and Preparing Audio Visuals (08)

Educational tools utilized encompassed flipboards, digital presentations, interactive workshops, quizzes, case presentations, and group assignments. Some lectures were streamed online but had also been pre-recorded serving as back-up for internet disruption. Lecturers from outside Malawi participated online to answer questions and join discussions.

### Coordinating the Program (09)

Coordination of the program was facilitated by a steering group comprising members from the organizing committee, supported by a Malawian medical secretary. Each day of the course had designated coupled ‘day coordinators’ (one Malawian specialist, one Dutch specialist) responsible for organizing the specific content and educational framework. Additionally, an online chat group was established via a messaging platform, enabling regular digital meetings with the steering group and the entire faculty. Furthermore, each day coordinator created a dedicated online group where all the speakers/lecturers for that day were included.

### Evaluating the Program (10)

Training evaluation represents the final step in the successful implementation of training program. Daily questionnaires were distributed to 35 participants to evaluate lecture and session quality. These anonymous surveys utilized a 5-point Likert scale and provided space for additional comments. Participants could expand on their responses or offer feedback beyond the scale in the comment section. Pre- and post-course questionnaires were also administered to participants and lecturers. Two months later, a ‘back to work’ questionnaire was sent to trainees to assess the course’s impact on their clinical behaviour concerning the recognition, treatment, and referral of oncologic patients. All evaluation forms were securely stored on an online server hosted by University Utrecht, The Netherlands, and checked for data completeness.

The educational effectiveness of the course was evaluated using Kirkpatrick's four levels of evaluation [12]. This model assesses training programs based on four criteria: reaction, learning, behaviour, and results. For the evaluation of the first level of the Kirkpatrick model, satisfaction level of five variables was done including: general education quality, relevance of the course, mode of the delivery of training, quality and knowledge of facilitators, and the overall course organisation for each day. The second level evaluates the acquisition of knowledge, skills, attitude, confidence, and commitment, performed by checking the level of confidences of the participants in managing 16 types of cancer, leading a multidisciplinary team meeting, and providing palliative surgical care before and after the course. The third level assesses the application of acquired knowledge in participants’ work, for which a ‘back to work’ questionnaire was used. The fourth level measures the achievement of intended outcomes, including support and accountability, which could not be assessed. There was sent a questionnaire to all lecturers once during the course week.

### Impact and Costs

The participating residents of the course as well as the training faculty were asked whether attending or conducting this course had impact on their clinical duties. The total cost of organizing the course has been calculated by adding all the expenses.

### Statistics

For statistical analysis, the SPSS version 29 (IBM Corp. in Armonk, NY.) was used. For level 2 of the Kirkpatrick method, we calculated medians and interquartile ranges (IQR) for non-normal distributed data. An independent sample median test with Yates’s correction for continuity to compare pre- and post- course levels of confidence in treatment of several cancers was used. As the pre- and post-course data was completely anonymized, we were unable to use paired statistical tests.

### Ethics

No ethical clearance was required in this course evaluation. All participants permitted the use of their anonymized questionnaire results.

## Results

Out of 83 eligible surgical and gynaecological COSECSA residents in Malawi, a total of 49 (59%) residents signed up for the course. We selected 35 (42%) to participate (as this was our predetermined number of participants) with help of the national training site director. Among the 35 trainees, 28 (80%) were physically present for all 5 days of the course, whereas the other seven (20%) attended at least one lecture remotely. Most of the attending surgical residents were female (59%, 17/28). An additional 16 gynaecological trainees attended the gynaecology lectures, with four (25%) attending in person and 12 (75%) participating online. Only one trainee (4%) had completed a dedicated oncology rotation before. Out of the total number of 35 surgical trainees, 28 (80%) completed all daily surveys and pre- and post-course questionnaires.

### Evaluation Kirkpatrick Level 1: Reaction, Perception, and Relevance

**Fig. 1:**
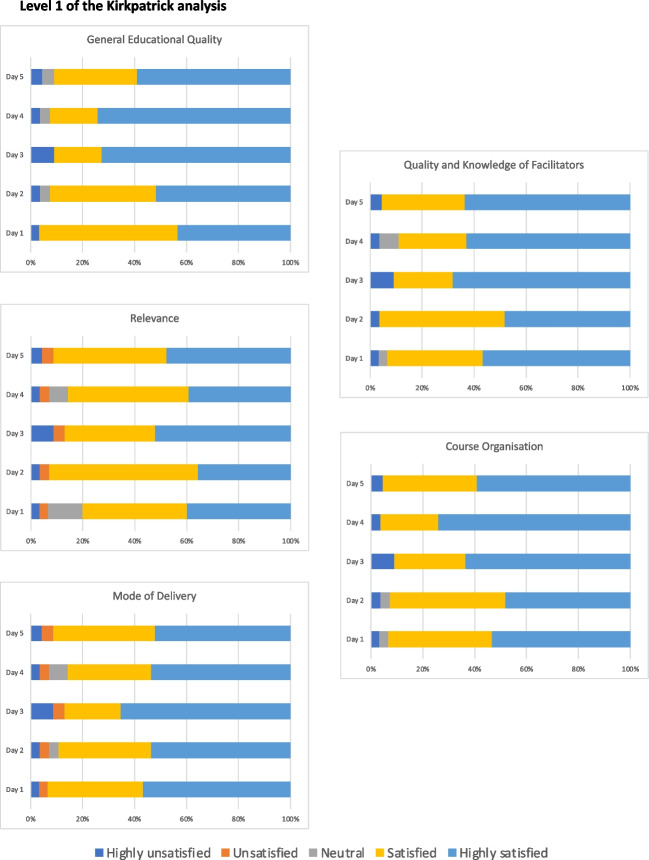
Bar graphs showing the responses of participants to aspects of the course related to level 1 of the Kirkpatrick analysis (satisfaction, engagement, and relevance). The *X*-axis notes the percentage of participants of each response on the 5-point satisfaction scale

Of the 28 trainees that completed all surveys, 24 (86%) of the respondents (*n* = 24/28) expressed a high satisfaction rate with the general educational quality for each of the five training days, indicating the training’s relevance and engagement with their jobs. The respondents (79%; *n* = 22/28) reported high satisfaction with the relevance of the course, mode of delivery of training, quality and knowledge of the facilitators, and overall course organization for each day (Fig. 1).

Furthermore, on open questioning, participants expressed their positive response towards the blended interactive learning approach (*n* = 10), the diversity of speakers from various countries (*n* = 6), and the multidisciplinary interaction with different specialists (*n* = 4). In terms of constructive feedback, 12 participants noted the absence of lectures on brain and bone cancers, which they hoped would have been included in the course. Another three suggested to organize more practical sessions in addition to the lectures. During the daily evaluations, participants expressed concerns about time management and interruptions during live lectures caused by internet connection problems. No complaints were recorded.

One participant scored the tick box ‘highly unsatisfied’ on all points every day, whereas the written feedback included only positive points amongst others ‘excellent updated data for us to forge the way forward in ‘LMIC’ and ‘none’ on the question ‘What do you wish to see improved on today’s course’. It is possible that this respondent inversely interpreted the 5-point scoring scale.

### Evaluation Kirkpatrick Level 2: Learning

**Fig. 2:**
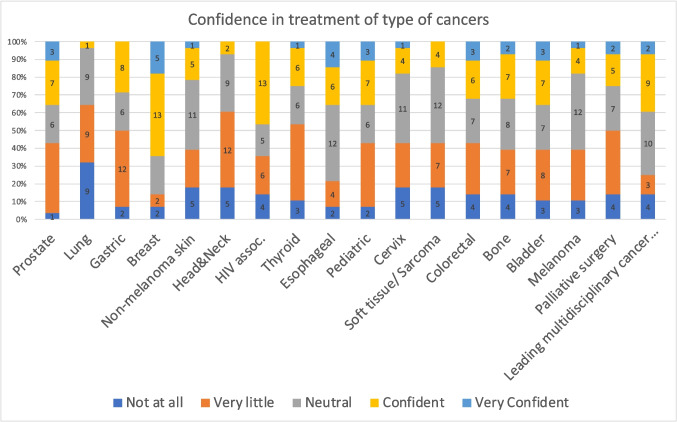
Pre-course confidence levels of treatment of different types of cancer

**Fig. 3:**
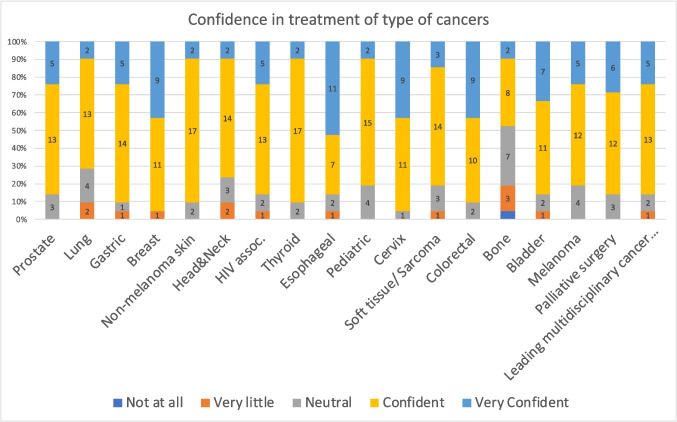
Post-course confidence levels of treatment of different types of cancer

**Fig. 4:**
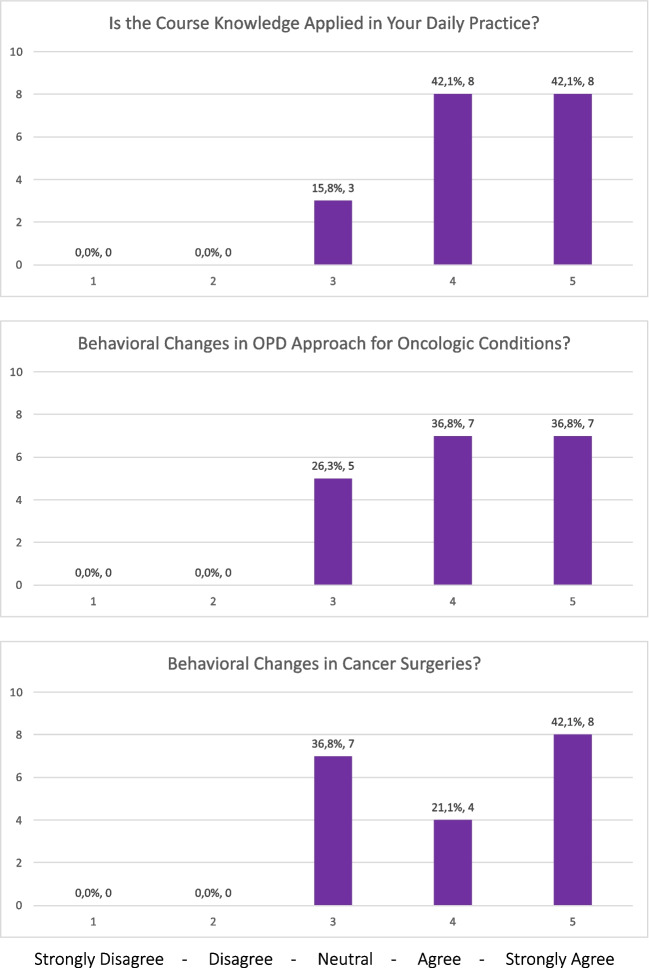
Results of back to work evaluation

The median (IQR) scores of each topic, of the participants who completed both pre- and post-course surveys, are shown in Table 2 and Figs. 2 and 3. In ten out of 18 topics (lung, gastric, head and neck, oesophageal, colorectal, urological cancers, paediatric oncology, cervical carcinoma, and HIV associated cancers), the students scored significantly higher (median) in the post-course survey, compared to the pre-course survey. In their daily practice, 16 out of 19 trainees (84%) reported using the newly acquired knowledge ‘often’ or ‘very often’. Additionally, 14 out of 19 (74%) trainees reported ‘often’ or ‘very often’ regarding implementing changes in the outpatient department for their patients with cancer, whilst 12 out of 19 (63%) trainees stated they ‘often’ or ‘very often’ changed their surgical approach in the theatre following the completion of this course (Fig. 4).

**Table 2:** Comparison of post and pre course median scores on the confidence levels of 18 cancers from the course surveys

Topic	Pre-test median (IQR)	Post-test median (IQR)	*P* value*
Prostate	3 (2–4)	4 (4–4)	*P* = ns
Lung	**2 (1–3)**	**4 (3–4)**	***P* < 0.05**
Gastric	**3 (2–4)**	**4 (4–4)**	***P* < 0.05**
Breast	4 (3–4)	4 (4–5)	*P* = ns
Non-melanoma skin	3 (2–3)	4 (4–4)	*P* = ns
Head and neck	**2 (2–3)**	**4 (4–4)**	***P* < 0.05**
HIV associated	**3 (2–3)**	**4 (4–4)**	***P* < 0.05**
Thyroid	2 (2–4)	4 (4–4)	*P* = ns
Oesophageal	**3 (3–4)**	**5 (4–5)**	***P* < 0.05**
Paediatric	**3 (2–3)**	**4 (4–4)**	***P* < 0.05**
Cervix	**3 (2–3)**	**4 (4–5)**	***P* < 0.05**
Soft tissue/sarcoma	3 (2–3)	4 (4–4)	*P* = ns
Colorectal	**3 (2–4)**	**4 (4–5)**	***P* < 0.05**
Bone	3 (2–3)	3 (3–4)	*P* = ns
Bladder	**2 (2–4)**	**4 (4–5)**	***P* < 0.05**
Melanoma	3 (2–4)	4 (4–4)	*P* = ns
MDP	**3 (2–3)**	**4 (4–4)**	***P* < 0.05**
Palliative	3 (2–4)	4 (4–5)	*P* = ns

Levels of confidence ranging from 1 (not at all) to 5 (very confident) (see Fig. 2)

### Impact and Costs

Out of the 35 selected participants, 28 (80%) were absent from their hospitals for five consecutive days. The attending trainees had to balance coursework with evening or night shifts to fulfil clinical duties. During the course, eight out of 13 national and international lecturers (62%) delayed their outpatient department work, and six out of 13 (40%) cancelled surgeries to deliver lectures. The total cost of organizing the course was €20,877 EUR, including venue rental, food, (international) travel, and lodging. When divided among the attendees, the cost per trainee per day was €119. Most expenses were allocated to the venue, food, and travel, course materials, promotion, local costs, and hosting the digital course platform. International lecturers mostly covered their own travel costs and lodging.

## Discussion

To our knowledge, this paper is the first to describe the co-creation and evaluation of a surgical oncology course in SSA through a collaborative effort between colleagues from both LMIC and HIC. This collaborative approach contributes to addressing the need for regional training and education and establishes a valuable framework for future initiatives in surgical oncology education. In this paper, we presented the results of our training following the steps of the Kirkpatrick evaluation method [12]. The feedback from participants indicated high satisfaction and increased confidence and knowledge application in clinical practice. These findings underscore the demand and necessity for a comprehensive oncology course and structured education for surgical healthcare workers in Malawi. This study sets a valuable precedent for future courses in similar settings. The growing burden of oncological conditions like colorectal and breast cancer, coupled with limited training opportunities for residents, prompted the surgical department of Kamuzu University of Health Sciences in Blantyre, Malawi, to recognize the necessity for a collaborative development of a course in surgical oncology.

An innovative aspect of our surgical oncology course was its co-creation and context-based design, which brought together a diverse faculty comprising both national and international experts in the field. By situating the course in Malawi, close to the participants' own working environment, it offered relevant and realistic clinical examples. This approach may not only have facilitated active participation of surgical residents but also promoted the development of a national and international network of expertise in surgical oncology. This setting and approach, distinct from other international courses organized in high-income settings, may have provided an advantage that potentially enhanced the overall learning experience.

### Limitations

While the feedback was overall positive in the Kirkpatrick level 1 and 2 evaluations, the questionnaires were short, and therefore, available data was relatively limited. Furthermore, the anonymous questionnaires were not numbered in our aim for anonymous data collection, but this hindered our statistical analysis in pairing the pre- and post-course evaluations. The course may benefit from a more robust evaluation strategy in which questionnaires are paired and the lecturer’s feedback is considered as well.

One respondent possibly inversely interpreted the 5-point scoring scale as he/she scored the tick box ‘highly unsatisfied’ on all points every day, whereas the written feedback included only positive points. This questionnaire has been included in the statistical analysis, as the explanation ‘inverse interpretation’ is still an assumption. This example shows the importance of outlining the evaluation even more clearly to the participants of future courses and considering the use of plus-and-minus rating scales instead of the 5-point scoring scale.

### Future Courses

Equal distribution of participants from all regions of Malawi and all surgical workforces will be ensured by collaboration with COSECSA and hopefully from other involved healthcare workers in the field like clinical officers and medical students. Previous analysis has shown that training non-physician clinicians such as clinical officers may improve surgical outcomes [13]. The absence of surgical residents from their work during the week may have potentially been harmful. Allowing only a portion of surgical residents from each medical facility to attend the course can mitigate this potential harm. Due to the venue's limited Wi-Fi, a stable connection for online sessions was not possible, leading to disruptions in live digital presentations and online lectures. To address this, pre-recorded presentations were used, which proved more effective due to fewer technical issues and allowed presenters to interact with the audience post-presentation. This approach, complemented by the coordinators' familiarity with the content, enhanced audience engagement. Our experience suggests that live presentations are preferable for educational dynamics, but when not feasible, pre-recorded content with digital presence of the presenter, is a viable alternative.

Ideally oncological diseases should be part of the standard curriculum of surgical and gynaecological residents, and practical training is a cornerstone of surgical oncology. We have shown that a short introduction training course is feasible in Malawi, where the residents enjoyed and learned from the content of this course.

In conclusion, incorporating surgical oncology training into the curriculum for residents in low-resource settings is an important factor in the preparation for the anticipated rise in the number of cancer cases. In this manuscript, we demonstrated the co-creation and organization of a short oncology course in Malawi; its success may be attributed to its context-based approach; a combined faculty from low and high-income settings and integration into an existing surgical training program. The course is expected to have a positive educational impact, with preliminary evidence suggesting improvements in clinical confidence and behaviour. For the future, we expect continued professionalisation of the course with a more robust evaluation system and further structured assessments, including pre- and post-course examinations, as well as post-course follow-ups. Additionally, evaluating the impact of the course on oncology diagnosis and care at the institutional level would be beneficial for future iterations.

## Declarations

### Disclaimer

The funders had no role in the development of the course program, and in the outcome of the data collection, analysis, or writing of the manuscript, nor the decision to submit the manuscript for publication.
